# Multimodal Treatment with GEMOX Plus Helical Tomotherapy in Unresectable Locally Advanced Pancreatic Cancer: A Pooled Analysis of Two Phase 2 Studies

**DOI:** 10.3390/biom11081200

**Published:** 2021-08-12

**Authors:** Alessandro Passardi, Ilario Giovanni Rapposelli, Emanuela Scarpi, Francesco Giulio Sullo, Giulia Bartolini, Elisa Neri, Giulia Ghigi, Luca Tontini, Giorgio Ercolani, Manlio Monti, Silvia Ruscelli, Laura Matteucci, Martina Valgiusti, Giovanni Luca Frassineti, Antonino Romeo

**Affiliations:** 1 Department of Medical Oncology, IRCCS Istituto Romagnolo Per lo Studio dei Tumori “Dino Amadori”—IRST, 47014 Meldola, Italy; alessandro.passardi@irst.emr.it (A.P.); francesco.sullo@irst.emr.it (F.G.S.); giulia.bartolini@irst.emr.it (G.B.); manlio.monti@irst.emr.it (M.M.); silvia.ruscelli@irst.emr.it (S.R.); laura.matteucci@irst.emr.it (L.M.); martina.valgiusti@irst.emr.it (M.V.); luca.frassineti@irst.emr.it (G.L.F.); 2 Unit of Biostatistics and Clinical Trials, IRCCS Istituto Romagnolo Per lo Studio dei Tumori “Dino Amadori”—IRST, 47014 Meldola, Italy; emanuela.scarpi@irst.emr.it; 3 Radiotherapy Unit, IRCCS Istituto Romagnolo Per lo Studio dei Tumori “Dino Amadori”—IRST, 47014 Meldola, Italy; elisa.neri@irst.emr.it (E.N.); giulia.ghigi@irst.emr.it (G.G.); luca.tontini@irst.emr.it (L.T.); antonino.romeo@irst.emr.it (A.R.); 4 General and Oncologic Surgery Unit, Morgagni-Pierantoni Hospital, AUSL Romagna, 47121 Forlì, Italy; giorgio.ercolani@auslromagna.it; 5 Department of Medical and Surgical Sciences, University of Bologna, 40126 Bologna, Italy

**Keywords:** pancreatic cancer, multimodal treatment, GEMOX, tomotherapy

## Abstract

In locally advanced pancreatic cancer (LAPC), the combination of chemotherapy and radiotherapy is a widely used treatment option. We performed a pooled analysis, including an exploratory analysis for prognostic and predictive factors, of two phase 2 trials including 73 patients with LAPC, treated with gemcitabine and oxaliplatin (GEMOX) and hypofractionated tomotherapy. With a median follow-up of 36 months (range 1–65), median progression-free (PFS) and overall survival (OS) were 10.2 (95% confidence interval [CI] 7.8–13.2) and 14.3 (95% CI 12.0–18.1) months, respectively. The overall resectability rate was 23.3% (95% CI 13.6–33.0), and the R0 resection rate was 13.7% (95% CI 5.8–21.6). In the multivariate analysis, ECOG performance status (PS) 0 and low levels of CA 19–9 were associated with improved OS and PFS. Concerning OS, log(CA19–9) resulted in a hazard ratio (HR) of 1.20 (95% CI 1.02–1.42), *p* = 0.027. For ECOG PS 0, HR was 1.00; for PS 1, HR was 2.69 (95% CI 1.46–4.96); for PS 2, HR was 4.18 (95% CI 0.90–19.46); *p* = 0.003. Low CA19–9 levels were also predictive for resection, with an odds ratio of 0.71 (95% CI 0.52–0.97), *p* = 0.034. In conclusion, GEMOX and hypofractionated radiotherapy is a treatment option in LAPC. Further studies are needed to identify differences in tumor biology, which may help to predict resectability and prognosis.

## 1. Introduction

Pancreatic cancer is the seventh leading cause of cancer-related mortality, with a 5-year survival rate around 10% [1,2]. About one third of patients affected by this malignancy present radiological evidence of locally advanced unresectable disease. This condition is defined by the absence of distant metastases and by local artery involvement (interface between tumor and superior mesenteric artery or celiac axis > 180° of vessel wall circumference), occlusion, or distortion of the superior mesenteric vein or portal vein, thereby not allowing for a safe reconstruction, and aortic or nodal involvement beyond the field of resection [3]. In this setting, life expectancy is low, with a median overall survival (OS) lower than one year [1].

Chemotherapy remains the standard approach in locally advanced pancreatic cancer (LAPC), with regimens derived from the metastatic setting, such as FOLFIRINOX and gemcitabine + nab-paclitaxel, currently considered as privileged regimens. Indeed, a systematic review and meta-analysis showed a median survival of 24.2 months in first-line treatment of LAPC with FOLFIRINOX, longer than that reported with gemcitabine [4]. As for gemcitabine + nab-paclitaxel, a randomized phase 2 trial in LAPC showed a reduction of disease progression rate after 3 months compared with gemcitabine alone (25.4% vs. 45.6%), along with a higher response rate (27% vs. 5.3%) and a positive effect on progression-free survival (PFS; 7 vs. 4 months) [5]. Promising results have also come from other combination regimens, such as PAXG; indeed, a randomized phase 2 trial that evaluated the addition of cisplatin and capecitabine to gemcitabine and nab-paclitaxel reported a 1-year PFS rate of 58% (vs. 39% in the control arm) and an OS rate at 18 months of 69% (vs. 54% in the control arm) [6].

The role of chemoradiotherapy (CRT) remains controversial. In 1981, Moertel et al. demonstrated the superiority of 5-fluorouracil-based CRT over chemotherapy alone in the management of LAPC [7]. Despite the promising results of this study, the use of CRT has decreased over time as a consequence of contradictory outcomes from studies comparing this therapeutic strategy with gemcitabine alone [8,9]. Interest in CRT has grown after recent retrospective studies demonstrated that the addition of induction chemotherapy could improve survival in patients with LAPC [10,11]. The LAP07 trial showed an increase in disease control in patients treated with CRT following induction chemotherapy, but failed to demonstrate a significant benefit in survival [12].

The potential role of CRT has further grown after the spread of hypofractionated radiotherapy (RT), which allows for the delivery of higher doses of radiation to the tumor whilst reducing patients’ time off full-dose chemotherapy. However, sparing of normal tissues is essential for a good quality of life. Several studies have demonstrated that stereotactic body radiation therapy (SBRT) allows for good local control (approximately 80% at 1 year) but still poor survival rates, with most patients dying from metastatic disease [13,14,15,16,17,18,19].

We have previously reported results of two phase 2 trials that exploited a “sandwich” strategy in pancreatic cancer patients with locally advanced, inoperable disease—both treatment plans, albeit slightly different in the number of chemotherapy cycles and the radiation dose and schedule, included an induction chemotherapy with gemcitabine plus oxaliplatin (GEMOX), then hypofractionated RT, then again the same chemotherapy schedule [20,21].

We here report the results of a pooled analysis of these two trials: in addition to an evaluation of the efficacy and safety of the association of GEMOX and hypofractionated RT in LAPC, we have performed an exploratory analysis that aimed to identify prognostic and predictive factors for conversion to resectability in this setting.

## 2. Materials and Methods

The acquisition of primary data took place between November 2004 and August 2016. For details on study protocols, please refer to the original publications [20,21].

### 2.1. Patient Eligibility

Main inclusion criteria of both trials included: histologically or cytologically confirmed diagnosis of pancreatic cancer, inoperable disease (by radiological and surgical evaluation), age ≥18 years and ≤75 years, life expectancy ≥12 weeks, ECOG performance status (PS) 0–2, and normal organ and marrow function. The principal exclusion criteria were: prior systemic therapy or radiotherapy, distant metastases, pregnancy or breast-feeding, active brain or leptomeningeal disease, currently active second malignancies (except non-melanoma skin cancers), and severe concurrent disease in the judgement of the investigator.

### 2.2. Treatment Plan

All patients were treated with a GEMOX regimen: gemcitabine (GEM) 1000 mg/m^2^ on day 1, and oxaliplatin (OX) 100 mg/m^2^ on day 2, every two weeks.

**Figure 1:**
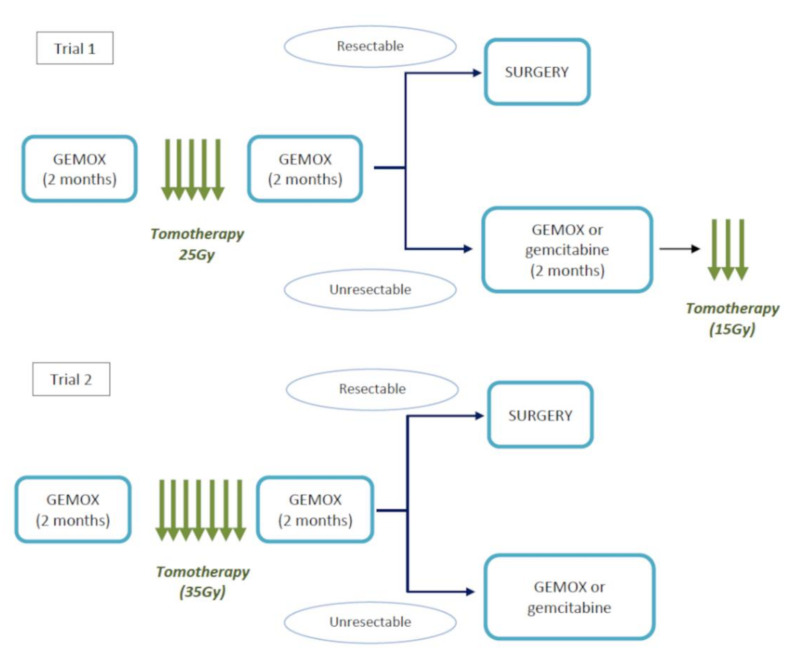
Treatment strategy with chemotherapy (GEMOX) and tomotherapy. In trial 1 [20], patients received chemotherapy with gemcitabine + oxaliplatin (GEMOX) for 2 months, then radiotherapy (RT; 25 Gy in 5 fractions, starting 15 days after last cycle), then GEMOX again for 2 months and were subsequently evaluated for surgery. Potentially resectable patients were submitted to surgery, while unresectable responders received chemotherapy for an additional 2 months with GEMOX or gemcitabine alone, at the discretion of the investigator, and further RT (15 Gy in 3 fractions). In trial 2 [21], patients received GEMOX for 2 months, then RT (35 Gy in 7 fractions, starting 15 days after the 4th cycle), then GEMOX for an additional 2 months and were evaluated for surgery. Potentially resectable patients underwent surgery, while unresectable responders received further cycles of GEMOX or gemcitabine alone, at the discretion of the investigator, as maintenance therapy.

In trial 1 [20], patients underwent RT (25 Gy in 5 fractions) 15 days after the 3rd–4th chemotherapy cycle. Patients then received further 3–4 cycles of GEMOX and were evaluated for surgery. Potentially resectable patients were submitted to surgery, while unresectable responders received 3–4 more cycles of GEMOX (or 2 cycles of GEM 1000 mg/m^2^ on days 1–8–15 every 28 days, at the discretion of the investigator) and further RT (15 Gy in 3 sessions). In trial 2 [21], RT had a different schedule (35 Gy in 7 fractions for 9 consecutive days) and was performed 15 days after the 4th cycle of GEMOX, then patients received 4 additional cycles of GEMOX and were evaluated for surgery. Potentially resectable patients underwent surgery, while unresectable responders received further cycles of GEMOX or GEM alone as maintenance, at the discretion of the investigator (Figure 1).

In both trials, RT was performed using helical tomotherapy. Patients were initially scanned on a contrast enhanced computed tomography (CT) simulator using 3 mm slice thickness to define the treatment plan according to tumor mass, lymph nodes, and organs at risk. As with other intensity-modulated radiation therapy (IMRT) techniques, inverse planning for tomotherapy required comprehensive contouring of organs at risk, as well as the identification of the regions to be treated—the gross tumor volume (GTV), including the tumor mass; the clinical tumor volume (CTV) 1, containing lymph nodal metastases; and the CTV2, which refers to regional lymph nodes (at risk of microscopic diffusion). Before each treatment fraction, patients underwent daily scanning and were repositioned after co-registration of the images with the simulation CT scan. Liver, kidneys, small bowel, stomach and bone marrow were found to be organs at risk. Treatment was delivered by helical tomotherapy.

In both trials, the total doses (35 Gy in 7 fractions and 25 Gy in 5 fractions) were prescribed to the 60–70% isodose line of the maximum dose covering the CTV1, with an increasing inhomogeneous dose within the tumor of up to 49 Gy in 7 fractions and 37.5 Gy in 5 fractions.

In both trials, no adjuvant treatment was considered for patients who underwent resection.

Chemotherapy toxicity was managed according to institutional routine clinical practice. As for RT-related toxicity, the occurrence of a grade ≥3 enteritis, gastritis, malabsorption, or nausea caused a 1-day postponement of treatment; in the event of persistence of one of the above toxicities for at least 3 consecutive days, the protocol contemplated RT discontinuation.

### 2.3. Statistical Considerations

The aim of this exploratory analysis was to evaluate prognostic factors in patients with LAPC recruited in two prospective phase 2 studies with similar eligibility criteria and treatment. Efficacy and toxicity analyses were performed on all patients who received at least one dose of study treatment. Resectability was defined as the absence of: superior mesenteric artery and celiac trunk encasement, invasion of aorta or inferior vena cava, occlusion of mesenteric or portal vein, and distant metastases. Objective tumor response was assessed using RECIST (Response Evaluation Criteria in Solid Tumors). The objective tumor response rate (ORR) was defined as the proportion of the intention-to-treat (ITT) population showing a complete or partial response, if confirmed ≥4 weeks later.

Overall survival (OS) was calculated as the time elapsed between the date of registration and the date of death due to any cause or the date of last follow-up. Progression-free survival (PFS) was calculated as the time elapsed between the date of registration and the date of the first observation of documentation of objective disease progression or death due to any cause, whichever occurred first, or last tumor evaluation.

Descriptive statistics were reported as proportions, median values, and ranges. PFS and OS probabilities were estimated using Kaplan–Meier method and their 95% confidence intervals (95% CI) were computed using the Greenwood method. Statistical analyses were carried out with SAS Statistical software (version 9.4, SAS Institute, Cary, NC, USA).

The studies were performed in accordance with the principles of Good Clinical Practice and the ethical standards laid down in the Declaration of Helsinki. The protocols were approved by the local ethics committee and written informed consent was obtained from each patient.

## 3. Results

### 3.1. Patient Characteristics

We retrospectively analyzed a total of 73 patients treated within the two above-mentioned trials. Patient characteristics are listed in Table 1. The median age at the time of diagnosis was 67 years (range 40–75). Overall, 58.9% of patients were female, ECOG PS was 0 in 46 patients (63.0%) and 1–2 in 27 (37.0%). In total, 20 (27.4%) and 53 (72.6%) patients had stage II and III disease, respectively. Tumor site was the head of the pancreas in 48 patients (65.7%) and 30 patients had a biliary stent placed prior to treatment. CA19.9 was within the normal range in 21 patients (29.6%).

### 3.2. Treatment Administration

Patients received a total of 436 cycles of chemotherapy, with a median of six cycles per patient (range 1 to 12 cycles). Twenty-eight patients (70%) regularly completed treatment according to study protocol. Early interruption of treatment was reported for the remaining 22 patients (12 for early progression, 10 for patient’s or investigator’s decision). In particular, RT was not administered to 17 patients, and was administered at lower doses, as a palliative treatment, to two patients.

### 3.3. Toxicity

All 73 patients were evaluable for toxicity. Treatment was generally well tolerated and the most common adverse events are listed in Table 2. Grade 3–4 neutropenia and thrombocytopenia occurred in 10 patients (13.7%), whereas grade 3–4 anemia was reported in three patients (4.1%). The most frequent grade 3–4 non-hematological adverse events were hepatotoxicity (6.8%) and fever (5.5%).

In both trials, adverse reactions due to RT were tolerable and fully reversible. Moreover, no late toxicities, such as gastrointestinal ulcer or biliary or duodenal obstruction, were reported.

### 3.4. Efficacy

The overall resectability rate was 23.3% (95% CI 13.6–33.0), while the R0 resection rate was 13.7% (95% CI 5.8–21.6). None of the radically operated patients received any adjuvant treatment, whereas 37 patients after progression were treated with a second line, 5-fluorouracil-based chemotherapy. All but one of the 73 patients were evaluable for response, and the ORR was 27.4% (95% CI 17.2–37.6). After a median follow-up time of 36 months (range 1–65), the median PFS and OS were 10.2 (95% CI 7.8–13.2) and 14.3 (95% CI 12.0–18.1) months, respectively (Figure S1). The 1- and 2-year OS rates were 61% (95% CI 50–73) and 28% (95% CI 17–40), respectively (Table 3). More in detail, median OS was 31.1 months (95% CI 17.3–not reached) in resected patients and 12.2 months in unresected patients (95% CI 9.2–15.0; *p* = 0.002) (Table S1).

### 3.5. Analysis of Prognostic Factors

In the univariate analysis, higher CA19-9 level and PS 1 or 2 were associated with a shorter OS. For log(CA19–9), the hazard ratio (HR) was 1.25, with 95% CI 1.06–1.48; *p* = 0.009. For ECOG PS 0, median OS was 18.7 months (95% CI 13.6–31.1) and the HR was 1; for ECOG PS 1, median OS was 11.4 (95% CI 8.4–14.3) and the HR was 2.55 (95% CI 1.43–4.56); for ECOG PS 2, median OS was 3.5 months (95% CI 0.2–not reached) and the HR was 4.09 (95% CI 1.21–13.78); *p* = 0.002 (Table 4). No other variables have been found to have a prognostic value for OS.

For PFS, the same variables (CA19-9, PS) were of prognostic value in the univariate analysis. For log(CA19–9), the HR was 1.21 with a 95% CI 1.03–1.41 (*p* = 0.016). For ECOG PS 0, median PFS was 13.4 months (95% CI 9.3–15.9) and the HR was 1; for ECOG PS 1, median PFS was 7.8 (95% CI 6.0–10.0) and the HR was 2.19 (95% CI 1.27–3.78); for ECOG PS 2, median PFS was 1.7 months (95% CI 0.2–not reached) and the HR was 2.35 (95% CI 0.71–7.75); *p* = 0.013 (Table 5). No other variables have been found to have a prognostic value for PFS.

Multivariate analysis, including also age and gender, confirmed the prognostic value of CA19-9 and PS for both OS and PFS. Concerning OS, log(CA19–9) resulted in a HR of 1.20 (95% CI 1.02–1.42), *p* = 0.027. For ECOG PS 0, HR was 1.00; for ECOG PS 1, the HR was 2.69 (95% CI 1.46–4.96); for ECOG PS 2, the HR was 4.18 (95% CI 0.90–19.46); *p* = 0.003. As for PFS, log(CA19–9) resulted in a HR of 1.18 (95% CI 1.01–1.37), *p* = 0.039. For ECOG PS 0, the HR was 1.00; for ECOG PS 1, the HR was 2.42 (95% CI 1.36–4.30); for ECOG PS 2, the HR was 2.80 (95% CI 0.61–12.89); *p* = 0.008 (Table 6).

### 3.6. Analysis of Predictive Factors

We also performed an analysis that aimed to identify factors associated with a higher probability of undergoing resection. CA19–9 was the only predictive factor for resection—for log (CA19-9), the odds ratio (OR) was 0.71 (95% CI 0.52–0.97), *p* = 0.034 (Table 7). No other variables were associated with a different probability of resection after CRT.

## 4. Discussion

The present study involved a pooled analysis of two phase 2 trials of a multimodal treatment (GEMOX + helical tomotherapy) in LAPC [20,21]. In addition to the evaluation of efficacy and safety, we performed an exploratory analysis to identify prognostic and predictive factors for conversion to resectability in this setting.

Treatment of LAPC usually relies on the same chemotherapy regimens of metastatic disease. The addition of RT aims to increase survival and chances of resectability, and the usually recommended strategy is based on an induction chemotherapy followed by consolidation CRT or SBRT in non-progressing patients [17,22]. The evolution of chemotherapy regimens and radiation techniques is crucial in order to increase both resectability and survival. Indeed, several reports have demonstrated a survival advantage in resected patients with LAPC [23,24,25]. Nevertheless, the optimal chemotherapy and RT regimens have not been defined.

Intensive chemotherapy regimens, such as gemcitabine + nab-paclitaxel, FOLFIRINOX, and PAXG, are currently available for LAPC [4,5,6]. Although the optimal therapy schedule has not been defined, the use of multi-agent chemotherapy before SBRT correlates with negative margins upon resection [26]. Thus, the use of intensive regimens has been proposed in a multimodal strategy with RT [25] and a retrospective study showed that the intensification of induction chemotherapy before CRT improves PFS in LAPC, although failing to show an advantage in survival [27].

Here, we propose the use of GEMOX in a “sandwich” strategy, i.e., both before and after SBRT; a previous study that analyzed different combination regimens of SBRT and chemotherapy in LAPC reported that chemotherapy after SBRT may improve OS and PFS [28]. GEMOX has already been proposed in a multimodal treatment for LAPC [27,29,30], with promising results. More specifically, a phase 2 trial that evaluated a CRT regimen with 5-fluorouracil infusion and weekly oxaliplatin after four cycles of GEMOX showed median OS and PFS of 12.6 and 9.4 months, respectively [30]. Here, we report a median OS of 14.3 months and a PFS of 10.2 months, which were obtained with the same doublet regimen, but taking advantage of SBRT instead of CRT.

The use of SBRT has several advantages compared with conventionally fractionated RT or CRT, and this is a promising option as a multimodality therapeutic strategy for LAPC [15]. SBRT has a decreased treatment time, which allows for the delivery of local treatment, minimizing interruptions of systemic therapy, with the potential improvement of treatment outcome [17]. Moreover, patients treated with SBRT showed a better local control compared to CRT [31] and freedom from local progression was correlated with OS [15]. A retrospective review of a large national database showed that SBRT was associated with superior OS in comparison with conventionally fractionated RT for LAPC [32], and a systematic review and meta-analysis showed an advantage in 2-year OS with decreased acute grade 3/4 toxicity [33]. In the present study, adverse reactions due to RT were tolerable and fully reversible, with no late toxicities reported. Indeed, helical tomotherapy allows for a dose escalation of the internal target along with acceptable dose to the surrounding organs at risk [34]. Dose escalation in the target volume is of paramount importance, since pooled analyses suggest a dose response for tumor control probabilities with SBRT [35], and an association between radiation dose and resectability has been shown [26].

Here, we report an overall resectability rate of 23.3%, with a radical resection rate of 13.7%; it is worth noting that these results have been obtained with a doublet chemotherapy regimen (GEMOX) and SBRT. Studies about induction with FOLFIRINOX and subsequent SBRT in LAPC reported radical resections in 12–24% of patients [18,25,36], whereas a phase 2 trial with the addition of cetuximab to gemcitabine and oxaliplatin, as well as CRT with capecitabine in patients that did not reach resectable disease, reported 8.3% of radical surgical resections [37]. In our study, resected patients showed an advantage in survival (31.1 vs. 12.2 months in unresected); similarly, a study with FOLFIRINOX followed by SBRT showed a higher survival after resection (3-year OS 43% in patients who underwent surgery, 6.5% in unresected patients; *p* = 0.03) [25].

A crucial point in the management of LAPC is the selection, through the identification of prognostic and predictive factors, of patients with more chances to benefit from a multimodal treatment, including surgery for responsive patients. Indeed, patients who respond to chemotherapy probably have a favorable disease biology and should be selected for more aggressive upfront management and surgery, while patients with resistant disease should be spared from high-risk surgery [38]. In the present study, we identified baseline CA19-9 and ECOG PS as prognostic factors and CA19-9 as the only predictor for resection. Notably, the prognostic and predictive value of CA19-9 did not emerge when CA19-9 was analyzed as a dichotomic variable (lower or higher than the upper limit of normal), but resulted upon analysis as a continuous variable (where CA19-9 values have been expressed on a logarithmic scale) (Table 4, Table 5 and Table 7). Indeed, the analysis of CA19-9 as a continuous variable allowed us to avoid loss of meaningful information, even with a small sample size. Our findings are consistent with other reports—the role of baseline CA19-9 as a prognostic factor in LAPC has already been demonstrated in several reports with different treatment strategies [39,40,41]. Interestingly, CA19-9 decline during treatment also has a prognostic role, and has been suggested as a criterion for selection of patients that can benefit from resection after primary chemotherapy [23]. The predictive value of baseline CA19-9 value may reflect a subset of patients with a more favorable disease biology, i.e., patients with a lower baseline CA19-9 level that have a higher probability of receiving a benefit from the multimodal treatment and obtaining a conversion to a resectable status after treatment. As for PS, an ECOG PS > 1 has been described as an independent factor decreasing the probability of resection in LAPC treated with FOLFIRINOX [40]. Of note, baseline PS has been shown also as a predictor of toxicity in LAPC treated with induction chemotherapy followed by CRT [42]. On the other hand, we did not report a prognostic or predictive role for baseline values of inflammation-related indexes such as neutrophil-to-lymphocyte ratio (NLR), platelet-to-lymphocyte ratio (PLR), and systemic immune-inflammation index (SII). Instead, a decreased survival rate has been previously reported in patients with higher NLR [43,44]. However, it should be noted that these studies were based on different therapeutic strategies compared with the present work, thus we cannot exclude a different prognostic performance of the same index according to the treatment used. On the other hand, our report of a lack of prognostic role of PLR is consistent with other reports [44].

A point of strength of the present work is the inclusion of patients with locally advanced disease only, while other studies of multimodal treatment of pancreatic cancer often include this peculiar setting together with borderline resectable disease. On the other hand, this study has some limitations: firstly, the difference in treatment schedule between the two trials analyzed (number of chemotherapy cycles, dose and fractions of RT fractions); secondly, the sample analyzed (*n* = 73) may be underpowered to detect differences in some prognostic or predictive factors; thirdly, the two trials have been designed before the appearance of new, more intensive chemotherapy regimens in locally advanced disease.

Indeed, future improvements in the treatment of LAPC may derive from the integration in a multimodal strategy of more intensive regimens, such as FOLFIRINOX, gemcitabine + nab-paclitaxel, and PAXG, together with the use of targeted agents and technical advancements in RT, including the possibility to deliver intraoperative RT [45]. Furthermore, fundamental advancements in the identification of prognostic or predictive factors may derive not only from new circulating biomarkers [46], but also from radiomics [47].

In conclusion, GEMOX plus helical tomotherapy is a feasible multimodal strategy in LAPC, with a good safety profile and promising results in terms of survival and resection rate. Further studies are warranted in order to define the optimal combination of chemotherapy and RT. The integration of hypofractionated RT with potentially more active regimens, currently available in the management of LAPC, is worthy of investigation in this setting. Furthermore, the identification of prognostic and predictive biomarkers is crucial in order to identify differences in tumor biology and to identify optimal candidates for surgical resection, thus allowing an improvement in disease management and treatment outcomes.

## Figures and Tables

**Table 1:** Patient characteristics.

Clinical Feature	Trial 1 [20] (*n* = 33)	Trial 2 [21] (*n* = 40)	Total (*n* = 73)
	***n* (%)**	***n* (%)**	***n* (%)**
**Age (years)**, median value (range)	64 (40–75)	67 (41–75)	67 (40–75)
**Gender**			
Male	15 (45.4)	15 (37.5)	30 (41.1)
Female	18 (54.6)	25 (62.5)	43 (58.9)
**ECOG PS**			
0	21 (63.6)	25 (62.5)	46 (63.0)
1	9 (27.3)	14 (35.0)	23 (31.5)
2	3 (9.1)	1 (2.5)	4 (5.5)
**Histological classification**			
Adenocarcinoma	28 (84.8)	35 (87.5)	63 (86.3)
Mucinous adenocarcinoma	-	2 (5.0)	2 (2.7)
Carcinoma	-	3 (7.5)	3 (4.1)
Not available (CA19–9 > 200 U/mL)	5 (15.2)	-	5 (6.9)
**Stage**			
IIA	3 (9.1)	3 (7.5)	6 (8.2)
IIB	6 (18.2)	8 (20.0)	14 (19.2)
III	24 (72.7)	29 (72.5)	53 (72.6)
**Tumor site**			
Head	23 (69.7)	25 (62.5)	48 (65.7)
Body	8 (24.2)	13 (32.5)	21 (28.8)
Tail	2 (6.1)	2 (5.0)	4 (5.5)
**Biliary stent**			
No	21 (63.6)	22 (55.0)	43 (58.9)
Yes	12 (36.4)	18 (45.0)	30 (41.1)
**CA 19–9 baseline**			
<37 U/mL (UNL)	11 (33.3)	10 (26.3)	21 (29.6)
≥37 U/mL	22 (66.7)	28 (73.7)	50 (70.4)

ECOG PS, Eastern Cooperative Oncology Group performance status; UNL, upper normal limit.

**Table 2:** Maximum toxicity in 73 patients treated with GEMOX + tomotherapy.

Adverse Event	Grade		
	1	2	3–4
	*n* (%)	*n* (%)	*n* (%)
Neutropenia	10 (13.7)	4 (5.5)	10 (13.7)
Febrile neutropenia	0	0	0
Leucopenia	1 (1.4)	2 (2.7)	0
Thrombocytopenia	2 (2.7)	16 (21.9)	10 (13.7)
Anemia	11 (15.1)	3 (4.1)	3 (4.1)
Fatigue	5 (6.8)	27 (37.0)	1 (1.4)
Fever	11 (15.1)	9 (12.3)	4 (5.5)
Weight loss	2 (2.7)	1 (1.4)	0
Pain	5 (6.8)	14 (19.2)	0
Hepatotoxicity	2 (2.7)	4 (5.5)	5 (6.8)
Peripheral neuropathy	10 (13.7)	7 (9.6)	1 (1.4)
Allergic reaction	2 (2.7)	3 (4.1)	1 (1.4)
Nausea/vomiting	8 (11.0)	32 (43.8)	2 (2.7)
Diarrhoea	5 (6.8)	8 (11.0)	2 (2.7)
Constipation	4 (5.5)	5 (6.8)	0
Stomatitis	1 (1.4)	0	0
Alopecia	0	0	1 (1.4)
Hyporexia	4 (5.5)	1 (1.4)	0
Dysgeusia	1 (1.4)	1 (1.4)	0
Rash	1 (1.4)	3 (4.1)	0
Other	5 (6.8)	1 (1.4)	1 (1.4)

**Table 3:** Efficacy measures.

Outcome	Trial 1 [20] (*n* = 33)	Trial 2 [21] (*n* = 40)	Total (*n* = 73)
**Median follow-up** (months)	19	50	36 (range 1–65)
**PFS** (months), median (95% CI)	11.4 (7.8–13.4)	9.3 (6.2–14.9)	10.2 (7.8–13.2)
**OS** (months), median (95% CI)	13.6 (11.8–18.1)	15.8 (8.2–23.4)	14.3 (12.0–18.1)
1-year OS (95% CI)	63% (46–81)	59.2% (43.8–74.6)	61% (50–73)
2-year OS (95% CI)	21% (4–39)	32.3% (18.4–47.2)	28% (17–40)
**Best response**, *n* (%)			
CR	1 (3.0)	0 (0)	1 (1.4)
PR	14 (42.4)	5 (12.5)	19 (26.0)
SD	11 (33.3)	20 (50)	31 (42.5)
PD	6 (18.2)	15 (37.5)	21 (28.8)
Not evaluable	1 (3.0)	0 (0)	1 (1.4)
**Overall response rate (%)**	45.4%	12.5%	27.4%
**Resected patients, *n* (%)**	8 (24.2)	9 (22.5)	17 (23.3)
**Patients with R0 resection, *n* (%)**	5 (15.2)	5 (12.5)	10 (13.7)

CI, confidence interval; CR, complete response; OS, overall survival; PD, progressive disease; PFS, progression-free survival; PR, partial response; SD, stable disease.

**Table 4:** Univariate analysis of overall survival.

Clinical Feature	No. of Patients	No. of Events	Median OS (Months) (95% CI)	*p*	HR (95% CI)	*p*
**Log(NLR)**	71	52			1.36 (0.80–2.33)	0.258
**Log(PLR)**	71	52			1.24 (0.54–2.85)	0.607
**Log(SII)**	71	52			1.32 (0.79–2.19)	0.286
**Log(CA 19–9)**	71	52			1.25 (1.06–1.48)	0.009
**NLR**						
<3	46	33	15.8 (13.4–23.8)		1.00	
≥3	25	19	10.2 (6.6–17.3)	0.148	1.52 (0.86–2.68)	0.151
**PLR**						
<146	35	27	15.0 (9.9–23.6)		1.00	
≥146	36	25	13.9 (8.4–17.6)	0.481	1.22 (0.70–2.12)	0.481
**SII**						
<581,500	35	24	17.3 (12.0–23.6)		1.00	
≥581,500	36	28	13.9 (8.4–17.6)	0.447	1.24 (0.71–2.14)	0.449
**CA 19–9**						
<37	21	14	18.7 (9.9–24.4)		1.00	
≥37	50	38	13.6 (11.8–17.3)	0.373	1.32 (0.71–2.45)	0.375
**Age**						
<67	36	25	17.3 (9.4–24.4)		1.00	
≥67	37	28	13.4 (9.9–17.3)	0.339	1.30 (0.76–2.25)	0.341
**Gender**						
Male	30	22	13.4 (9.2–17.3)		1.00	
Female	43	31	15.3 (11.4–23.8)	0.514	0.83 (0.48–1.44)	0.514
**ECOG PS**						
0	46	28	18.7 (13.6–31.1)		1.00	
1	23	22	11.4 (8.4–14.3)		2.55 (1.43–4.56)	
2	4	3	3.5 (0.2–nr)	0.001	4.09 (1.21–13.78)	0.002
**Stage**						
IIA	6	3	18.7 (12.0–nr)		1.00	
IIB	14	9	16.7 (4.7–nr)		1.25 (0.34–4.64)	
III	53	41	13.4 (9.4–17.3)	0.171	2.15 (0.66–6.97)	0.182
**Tumour site**						
Head	48	35	13.8 (11.4–18.2)		1.00	
Body	21	15	15.3 (9.2–26.1)		0.87 (0.47–1.60)	
Tail	4	3	11.1 (2.7–nr)	0.443	1.94 (0.59–6.40)	0.456
**Biliary stent**						
No	43	30	15.3 (9.9–18.7)		1.00	
Yes	30	23	13.8 (8.4–23.4)	0.997	1.00 (0.58–1.73)	0.997

CI, confidence interval; ECOG PS, Eastern Cooperative Oncology Group performance status; HR, hazard ratio; NRL, neutrophil-to-lymphocyte ratio; nr, not reached; OS, overall survival; PLR, platelet-to-lymphocyte ratio; SII, systemic immune-inflammation index.

**Table 5:** Univariate analysis of progression-free survival.

Clinical Feature	No. of Patients	No. of Events	Median PFS (Months) (95% CI)	*p*	HR (95% CI)	*p*
**Log(NLR)**	71	57			1.05 (0.62–1.77)	0.863
**Log(PLR)**	71	57			0.87 (0.41–1.84)	0.720
**Log(SII)**	71	57			1.05 (0.66–1.68)	0.836
**Log(CA 19–9)**	71	57			1.21 (1.03–1.41)	0.016
**NLR**						
<3	46	37	12.3 (7.8–13.4)		1.00	
≥3	25	20	7.8 (5.4–15.9)	0.935	1.02 (0.59–1.78)	0.935
**PLR**						
<146	35	30	11.1 (7.8–14.1)		1.00	
≥146	36	27	9.3 (6.0–13.3)	0.894	0.96 (0.57–1.63)	
**SII**						
<581,500	35	28	11.1 (7.6–13.4)		1.00	
≥581,500	36	29	9.3 (6.0–13.3)	0.818	1.06 (0.63–1.79)	0.818
**CA 19–9**						
<37	21	15	11.1 (7.8–21.0)		1.00	
≥37	50	42	9.3 (6.0–13.3)	0.316	1.35 (0.75–2.44)	0.318
**Age**						
<67	36	28	12.3 (6.2–14.7)		1.00	
≥67	37	30	9.4 (6.4–13.4)	0.822	1.06 (0.63–1.78)	0.822
**Gender**						
Male	30	26	10.2 (6.5–13.4)		1.00	
Female	43	32	10.0 (6.4–15.5)	0.311	0.76 (0.45–1.29)	0.312
**ECOG PS**						
0	46	32	13.4 (9.3–15.9)		1.00	
1	23	23	7.8 (6.0–10.0)		2.19 (1.27–3.78)	
2	4	3	1.7 (0.2–nr)	0.010	2.35 (0.71–7.75)	0.013
**Stage**						
IIA	6	3	12.3 (11.1–nr)		1.00	
IIB	14	11	9.7 (4.7–14.7)		2.02 (0.56–7.27)	
III	53	44	8.4 (6.4–13.2)	0.291	2.43 (0.75–7.84)	0.311
**Tumour site**						
Head	48	36	11.1 (7.8–13.3)		1.00	
Body	21	18	8.4 (6.0–15.5)		1.02 (0.58–1.81)	
Tail	4	4	6.7 (0.9–nr)	0.538	1.78 (0.63–5.04)	0.547
**Biliary stent**						
No	43	34	10.0 (7.8–14.1)		1.00	
Yes	30	24	10.2 (6.0–13.4)	0.969	1.01 (0.60–1.70)	0.969

CI, confidence interval; ECOG PS, Eastern Cooperative Oncology Group performance status; HR, hazard ratio; NRL, neutrophil-to-lymphocyte ratio; nr, not reached; PFS, progression-free survival; PLR, platelet-to-lymphocyte ratio; SII, systemic immune-inflammation index.

**Table 6:** Multivariate analysis of progression-free and overall survival.

Clinical Feature	PFS		OS	
	HR (95% CI)	*p*	HR (95% CI)	*p*
**Log(CA 19–9)**	1.18 (1.01–1.37)	0.039	1.20 (1.02–1.42)	0.027
**ECOG PS**				
0	1.00		1.00	
1	2.42 (1.36–4.30)		2.69 (1.46–4.96)	
2	2.80 (0.61–12.89)	0.008	4.18 (0.90–19.46)	0.003
**Age**				
<67	1.00		1.00	
≥67	0.86 (0.49–1.48)	0.579	1.13 (0.65–1.99)	0.662
**Gender**				
Male	1.00		1.00	
Female	0.61 (0.34–1.10)	0.099	0.60 (0.33–1.10)	0.101

CI, confidence interval; ECOG PS, Eastern Cooperative Oncology Group performance status; HR, hazard ratio; OS, overall survival; PFS, progression-free survival.

**Table 7:** Association between patient characteristics and resectability.

Clinical Feature	OR (95% CI)	*p*
**Log(NLR)**	0.38 (0.12–1.20)	0.099
**Log(PLR)**	0.71 (0.16–3.05)	0.643
**Log(SII)**	0.52 (0.19–1.43)	0.205
**Log(CA 19–9)**	0.71 (0.52–0.97)	0.034
**NLR**		
<3	1.00	
≥3	0.35 (0.09–1.36)	0.128
**PLR**		
<146	1.00	
≥146	0.96 (0.32–2.94)	0.949
**SII**		
<581,500	1.00	
≥581,500	0.96 (0.32–2.94)	0.949
**CA 19–9**		
<37	1.00	
≥37	0.70 (0.22–2.25)	0.555
**Age**		
<67	1.00	
≥67	0.61 (0.20–1.82)	0.373
**Gender**		
Male	1.00	
Female	1.93 (0.60–6.23)	0.268
**ECOG PS**		
0	1.00	
1	0.38 (0.10–1.50)	
2	0.85 (0.08–8.89)	0.386
**Stage**		
IIA	1.00	
IIB	0.40 (0.05–2.89)	
III	0.23 (0.04–1.33)	0.233
**Tumor site**		
Head	1.00	
Body	0.40 (0.10–1.59)	
Tail	ne	0.433
**Biliary stent**		
No	1.00	
Yes	1.37 (0.46–4.10)	0.569

CI, confidence interval; ECOG PS, Eastern Cooperative Oncology Group performance status; OR, odds ratio; NRL, neutrophil-to-lymphocyte ratio; ne, not estimable; PLR, platelet-to-lymphocyte ratio; SII, systemic immune-inflammation index.

## Data Availability

Data are available, in anonymized form, upon reasonable request.

## Supplementary Materials

The following are available online at https://www.mdpi.com/article/10.3390/biom11081200/s1, Figure S1: Kaplan–Meier analysis of overall and progression-free survival of the whole study population; Table S1: Analysis of survival according to resection status.
