# Supplementary material for: Multimodal Treatment with GEMOX Plus Helical Tomotherapy in Unresectable Locally Advanced Pancreatic Cancer: A Pooled Analysis of Two Phase 2 Studies
Source: Biomolecules. 2021 Aug 12;11(8):1200. doi: 10.3390/biom11081200 (PMC8393939; doi:10.3390/biom11081200)
Supplement: Supplementary file 1 [file biomolecules-11-01200-s001.zip › biomolecules-1302453-supplementary.pdf]

biomolecules

MDPI

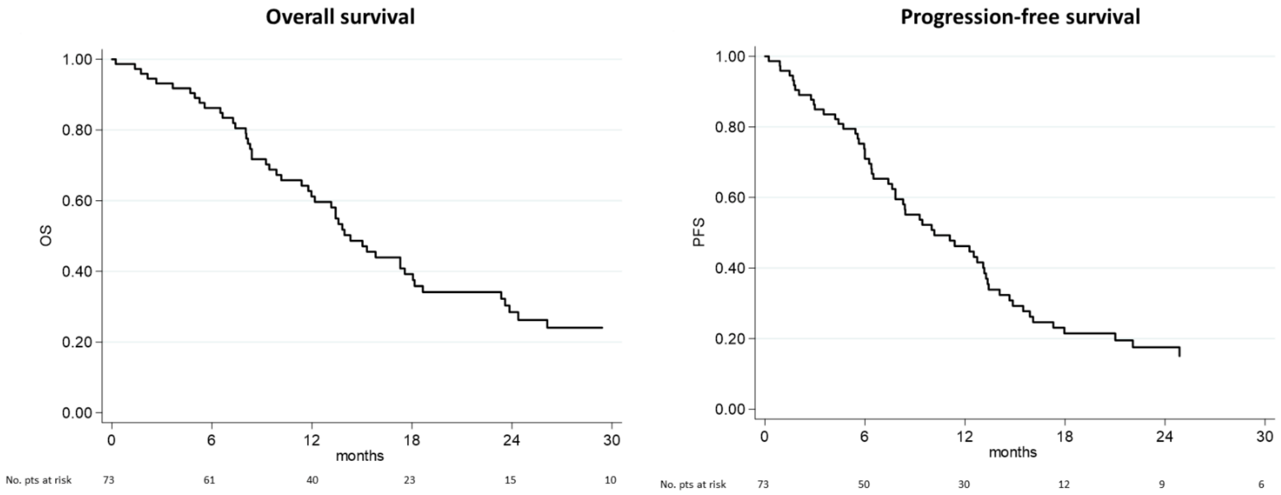

Figure S1. Kaplan-Meier analysis of overall and progression-free survival of the whole study population.

Table S1. Analysis of survival according to resection status.

	patients (*n*)	deaths (*n*)	1-year OS (95% CI)	2-year OS (95% CI)	OS (months) median (95% CI)	p
**Overall cohort**						
Unresected	56	44	49 (35–62)	20 (8–32)	12.2 (9.2–15.0)	
Resected	17	9	94 (83–100)	53 (27–79)	31.1 (17.3–nr)	0.002
**Patients that received RT**						
Unresected	40	28	61 (45–77)	26 (11–41)	13.8 (11.4–17.6)	
Resected	16	9	94 (82–100)	52 (25–78)	31.1 (17.3–nr)	0.029

CI, confidence interval; nr, not reached; OS, overall survival; RT, radiotherapy.
